# Design of a Multisensory Device for Tomato Volatile Compound Detection Based on a Mixed Metal Oxide—Electrochemical Sensor Array and Optical Reader

**DOI:** 10.3390/mi14091761

**Published:** 2023-09-12

**Authors:** Félix Meléndez, Ramiro Sánchez, Juan Álvaro Fernández, Yaiza Belacortu, Francisco Bermúdez, Patricia Arroyo, Daniel Martín-Vertedor, Jesús Lozano

**Affiliations:** 1 Industrial Engineering School, University of Extremadura, 06006 Badajoz, Spain; felixmv@unex.es (F.M.); jalvarof@unex.es (J.Á.F.); parroyoz@unex.es (P.A.); 2 Alianza Nanotecnología Diagnóstica ASJ S.L. (ANT), 28703 San Sebastián de los Reyes, Spain; yaizabelacortu@antd.eu (Y.B.); fran@grupotrafico.es (F.B.); 3 Centro de Investigaciones Científicas y Tecnológicas de Extremadura (CICYTEX), 06006 Badajoz, Spain; ramiro.sanchez@juntaex.es (R.S.); daniel.martin@juntaex.es (D.M.-V.)

**Keywords:** electronic nose, tomato maturity, metal oxide sensor, electrochemical sensor, optical reader, volatile organic compounds, *Botrytis cinerea*

## Abstract

Insufficient control of tomato ripening before harvesting and infection by fungal pests produce large economic losses in world tomato production. Aroma is an indicative parameter of the state of maturity and quality of the tomato. This study aimed to design an electronic system (TOMATO-NOSE) consisting of an array of 12 electrochemical sensors, commercial metal oxide semiconductor sensors, an optical camera for a lateral flow reader, and a smartphone application for device control and data storage. The system was used with tomatoes in different states of ripeness and health, as well as tomatoes infected with *Botrytis cinerea*. The results obtained through principal component analysis of the olfactory pattern of tomatoes and the reader images show that TOMATO-NOSE is a good tool for the farmer to control tomato ripeness before harvesting and for the early detection of *Botrytis cinerea*.

## 1. Introduction

Tomatoes are fruits belonging to the *Solanaceae* family that are harvested worldwide and are the second most widely consumed vegetable [1,2]. The global tomato market was USD 43.3 M in 2022, and it is projected to increase to USD 70.87 M by 2031 [3]. Furthermore, tomatoes are classified as a climacteric fruit (emitting ethylene volatile organic ripening phytohormone) with an intense flavor and aroma and, therefore, deteriorate rapidly after harvest and have increased susceptibility to microbial infection [4]. The USDA established the color maturity of tomatoes in six different stages: green (stage 1), breakers (stage 2: <10% of tomatoes’ surface are combined yellow-orange-red shades), turning (stage 3: 10–30% combined color), pink (stage 4: 30–60% combined color), light red (stage 5: 60–90% combined color), and red stage (stage 6: <90% red color) [5].

The aromatic profile of tomatoes depends on genetic factors and pre- and post-harvest conditions, whose interaction with taste and texture results in flavor perception [4,6]. The consumers and processed industry demand good aroma and flavor. For that reason, improving the qualities of these tomatoes has attracted increasing interest from growers and stakeholders [4]. Establishing the correct ripening phase is crucial to obtaining high-quality tomatoes and reducing tomato production losses, which are around 42%, mainly during post-harvest time [7]. Also, fungus pests are the main cause of pre- and post-harvest losses [7].

*Botrytis cinerea* is a silent latent threat that causes a devastating disease that affects important vegetables in fields and greenhouses and during the post-harvest time, causing economic losses of approximately USD 10–100 M per year [8]. It infects different organs (fruits, roots, vegetables, ornamental leaves, and flowers) of 1400 plant species, including tomato plants, either by injury after pruning and harvesting, transportation, or direct penetration, causing putrefaction events at all stages of the production chain [9]. Unfortunately, disease symptoms and the presence of conidia become visible to the naked eye later than host infection, as the pathogen remains latent for a long time inside the host and crop debris [10]. In addition, *B. cinerea* is increasingly resistant to conventional fungicides [11,12].

The increasing reduction in the use of chemical pesticides and fertilizer agents, climate changes, poor infrastructures, disease control, and post-harvest loss led to the need to encourage scientists to find new production practices and alternative control methods to help farmers increase yields and manage crops [11,12]. Also, plant pathogen detection conventionally relies on molecular technology that is complicated, time-consuming, constrained to centralized laboratories, and requires skilled staff. Different technologies are being developed under precision/smart agriculture to improve agriculture yields and fruit/vegetable qualities and reduce the use of chemicals (pesticides, fungicides, and fertilizers) to achieve a more sustainable economy and agriculture [12,13]. Some of these new technologies are related to Artificial Intelligence [13,14,15], Machine Vision [16], Multi-genetic OMICs [17], electronic noses (E-noses) [18,19,20], wearable sensors [21], rapid test as isothermal amplification of *Botrytis* DNA [22], visual immunoassays [23], and lateral flow devices (LFD), which are being applied in laboratory models and also in tomato crops [24,25].

Electronic noses (E-noses) are electronic devices that attempt to mimic the biological sense of smell [26]. For this purpose, E-noses include a set of gas sensors of different technologies, such as Metal Oxide Semiconductor (MOS) sensors [27,28,29], electrochemical (EC) sensors [30], conductive polymer (CP) sensors [31], quartz crystal microbalance (QMB) sensors [32], and surface acoustic wave (SAW) sensors [33], among others. In addition, some of these sensors may incorporate integrated circuits to provide their signal in digital format via I^2^C or SPI interfaces [34]. The data obtained by these sensors must be processed and used for some kind of pattern recognition or classification system, such as Principal Component Analysis (PCA) [35], Artificial Neural Network (ANN) [36], Fuzzy Logic (FL) [37], and Support Vector Machine (SVM) [38]. In recent years, E-noses have become devices successfully used in different applications and industries where volatile compounds or aromas play a key role. They have been used to discriminate defects in table olives [39], coffee [40], and beer [41]; in medicine for the detection of diseases by the breath [38] and in health monitoring [42]; for the measurement of air quality [43]; and for the detection of contaminated cork stoppers [44], among others. In agriculture, electronic noses have been successfully used for the detection of defects caused by microorganisms in strawberries [20], blueberries [45], oranges [46], kiwis [47], apple trees, bananas, and grapes [48]. This technology is a great tool for implementing smart agriculture to digitize crops, reduce costs, improve yields, and minimize environmental impact [49,50]. E-nose technology has been developed since the 1980s and has experienced vast progression from the technical to marketable stage [51]. Over the past few years, the reduction in the size of sensors and electronic noses has allowed E-noses to reduce in size, power consumption, and cost [52]. Thus, a portable, non-professional, fast, reliable, and real-time E-nose would be ideal for use in the field for the detection of crop diseases at asymptomatic stages and in other agri-food chains such as post-harvest.

More than 400 VOCs have been identified using Gas Chromatography–Mass Spectrometry (GC-MS) techniques in the tomato fruit [6], with only 40 presenting concentrations above one part per billion (ppb) [53,54]. Of these, only a few compounds (aldehydes, hydrocarbons, alcohols, ketones, furans, esters, nitrogen compounds, and heterocyclic sulfur and nitrogen compounds) contribute mainly to human aroma perception [53,54]. Some E-noses have been developed to detect tomato VOCs during fruit shelf life [55,56], for plant health monitoring [56,57,58,59], and for established biomarker control. Therefore, changes in the volatile profile may provide a marker for identifying infected tomato fruit with *B. cinerea* or other pathogens as other fruits [45,47,60].

In the present study, we design and develop the TOMATO-NOSE, a field-portable and user-friendly E-nose integrated with a lateral flow test reader and smartphone communication for the detection of the different tomato maturation stages and early *Botrytis cinerea* infection in crops or storage facilities by farmers. The TOMATO-NOSE uses an array of 13 different MOS and EC sensors to detect VOCs emitted by tomatoes during both events, and the reader confirms the presence of *Botrytis* infection by molecular antigen test. Principal Component Analysis (PCA) was used to investigate whether the E-nose could distinguish among different aromas. The main goal of this work is to study the TOMATO-NOSE discrimination capability throughout the ripeness states of the tomato and in the early detection of tomato fungus infection.

## 2. Materials and Methods

### 2.1. Samples

Roma tomatoes were purchased at the supermarket in good sanitary condition. Tomatoes were transported to the research center in ventilated storage trays to avoid any chemical changes at the commercial stage of maturation. They were immediately stored at 4 °C until the moment of the analysis. Tomatoes were selected at stages 4–6 of maturity.

#### 2.1.1. Samples of Bags and Air Containers

Polyamide/polyethylene bags from AlemPack Lda (Elvas, Portugal) were used. Open bags were closed by plastic food clips (Karells, Amazon, Moormerland, Germany) with a true tightness watertight double seal. The plastic containers were boxes 14 × 10 × 10 cm in size.

#### 2.1.2. Lateral Flow Test Device (LFD)

Two commercially available *Botrytis cinerea* LFDs (B-LFD) were used to detect soluble stable *Botrytis* antigens on tomato fruits: (1) the *Botrytis* Alert product from Mologic Ltd. (Thurleigh, UK), which uses *Botrytis* monoclonal antibody BC-12.CA4 [61], and (2) LOEWE^®^FAST Lateral Flow Kits from LOEWE Biochemica GmbH (Sauerlach, Germany).

BC-12.CA4 antibody recognizes an antigen, possibly a glycoprotein, with the antigenic binding site on L-rhamnose. The antigen is expressed from the first appearance of the germ tube during germination. It can be observed by immunofluorescence along the entire germ tube and at its tip, but not on the conidia. The test recognized *Botrytis* mycelium rather than the spore. No information about the antibody of LOEWE^®^FAST Lateral Flow Kits is available.

Positive and negative controls of *B. cinerea* antigen from grapevine mold were used (LOEWE Biochemica GmbH). These controls were manufactured for detection by the ELISA technique (Enzyme-Linked Immunosorbent Assay). In this work, the controls were adapted to be used in B-LFD. For that, they were reconstituted with 1 mL of a physiological buffer (NaCl 0.9%; PharmaSET, Toulouse, France) immediately before use instead of the sample buffer provided by the LFD manufacturer.

### 2.2. Botrytis Cinerea Infection

*Botrytis cinerea Persoon* 1794-CB303 strain (ref: 209731) from CECT (Colección Española de Cultivos Tipo, Paterna, Spain) was grown in PDA (Potato Dextrose Agar) with chloramphenicol medium until the fungus made spores. Potato infusion and dextrose promote luxuriant fungal growth. Adjusting the pH of the medium by tartaric acid to 3.5 inhibits bacterial growth. In total, 10^5^ spores.mL^−1^ were diluted in buffered peptone water (BPW). Uniform red tomatoes were selected and disinfected by immersion for 30 s in a 200 mg/mL sodium hypochlorite solution. After air-drying at room temperature, an incision was made using a sterilized stainless-steel rod (3 mm wide × 3 mm deep: two incisors per fruit). Twenty microliters of 10^5^ spores.mL^−1^ were inoculated in the incisions. The tomatoes were placed in a plastic transparent box kept at 25 °C and 100% humidity for 6–10 days to develop the fungus.

### 2.3. Description of the Prototype

The TOMATO-NOSE consists of two parts, which will be described in this section: the E-nose, which detects the tomato aroma, and the optical part for detecting *Botrytis cinerea* through vision and the LFD. This section also explains the communication protocol between the E-nose and the smartphone, the pneumatics of the device, the design of the cassettes, and the external design of the prototype.

#### 2.3.1. Electronic Nose Description

The E-nose contains an electronic circuit where the sensors are located, and a pneumatic circuit responsible for the distribution of gases to the sensors. The electronics have two printed circuit boards (PCB), which communicate via SPI bus and are placed parallel to each other and separated by a gas cell where the sensors are enclosed.

The main board is the bottom board. It is governed by a PIC32MM0256GPM048 microcontroller from Microchip Technology Inc. (Chandler, AZ, USA), which contains 256 KB of program memory, 32 KB of data memory, a frequency of 24 MHz, and several I^2^C, SPI, and UART communication modules, among other features. This board also has a Bluetooth low-energy module RN4871, also from Microchip, which communicates with the microcontroller via UART and operates in a transparent mode. In addition, it contains all the digital sensors of the electronic nose. These sensors communicate via the I^2^C bus with the microcontroller and return their data in digital format. All these sensors are MOS sensors except for one photoacoustic sensor. All of them are shown in Table 1. The main board also includes a cable-to-board connector to connect the solenoid valves, switched by the microcontroller. This circuit is powered by a TPS63001 power supply from Texas Instruments Inc. (Dallas, TX, USA), a DC-DC Buck-Boost converter with an output voltage of 3.3 V_DC_, and the MIC5504 linear regulator from Microchip, with an output voltage of 1.8 V_DC_.

The secondary board has the same microcontroller as the main board to facilitate communication between the two PCBs. Six sensors were placed on this board, all of them with industry-standard, cylindrical housing with a base diameter of 20 mm and a height of 16.5 mm. These six sensors are connected to the PCB via sockets, which allows an easy exchange of one sensor for another, either by sensor failure or by switching to another sensor with specificity to another gas. The secondary board sensors are shown in Table 1. All these sensors return analog signals, so it is necessary to amplify them using operational amplifiers like ADA4528-1ARMZ and ADA4666-2ARMZ from Analog Devices Inc. (Norwood, MA, USA), LT6014CS8/PBF from Linear Technology Corporation (Milpitas, CA, USA), and TLC27M2ACD from Texas Instruments. The amplified signals are connected to three analog-to-digital converters, Microchip’s MCP3424, each with four channels and 18-bit resolution, communicating with the microcontroller via an I^2^C bus. The two microcontrollers communicate via the SPI bus using a proprietary protocol created for this prototype.

#### 2.3.2. Multivariate Data Analysis

The measurements obtained with TOMATO-NOSE were processed with chemometric tools for interpretation, to identify outliers and to study the discrimination of potential groups. Exploratory data analysis was performed using PCA for visualization purposes. This type of analysis reduces the information provided by the E-nose to the minimum number of variables, called principal components. Principal components are linear combinations of the original response vectors [62]. The statistical tool used was MATLAB R2023a (The Mathworks Inc., Natick, MA, USA) with the PLS_Toolbox 9.1 (Eigenvector Research Inc., Wenatchee, WA, USA).

#### 2.3.3. Vision Part Description

**Figure 1:**
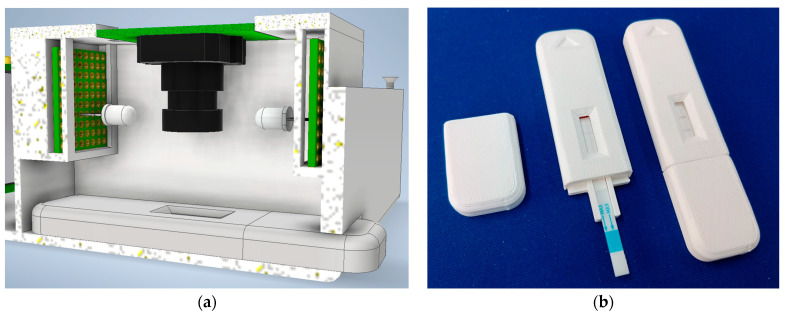
(**a**) Cross-section of the test image acquisition compartment; (**b**) holder cassettes designed to contain lateral flow test.

As for the vision part of the lateral flow strips, a Raspberry Pi 4 Model B with 2 GB of RAM and the Raspberry Pi OS version 11 (Bullseye) operating system was used. This Raspberry is powered by the PiJuice HAT, a portable uninterruptible power supply platform from the company Pi Supply (Nebra Ltd., Bells Yew Green, UK), which allows the Raspberry Pi 4 to be powered by a battery instead of an external AC adapter. The PiJuice HAT is powered by a 3.7 V_DC_, 1820 mAh Li-Po battery, although it contains a connector that allows installing higher-capacity batteries, from 5000 to 12,000 mAh. The PiJuice HAT has a B-type micro-USB connector, to which a smartphone charger can be connected to recharge the battery. This battery also powers the E-nose and the pneumatic pump, as the PiJuice HAT has 3.3 V_DC_ and 5 V_DC_ outputs. The OKdo 5 MP Camera, from OKdo Ltd. (London, UK), was used to take the photos. It has a 5 MPx sensor that provides color images of 2592 × 1944 px, with an aspect ratio of 4:3, a fisheye lens with adjustable manual focus, and its software allows storing the images in the chosen compressed format—in this case, lossless PNG [63]. The camera is connected to the main board via a flat cable connector to the Raspberry Pi 4’s CSI connector, allowing the Raspberry processor to access various functions of the camera, such as brightness and contrast control, as well as white balance and exposure time, and to perform the capture in a controlled manner. This camera was mounted on the device developed in this work with a focal length of 20 mm, placing the sensor vertically on the cassette. In order to control the illumination, the capture set was completed with two white Nichia Corporation (Tokushima, Japan) LEDs located on either side of the camera sensor at about 20 mm. As shown in Figure 1a, this system allows adequate illumination of the cassette, avoiding the projection of shadows on the strip, except those at its lateral ends, produced by the edges of the cassette window.

Once the image is captured, it is processed in an Octave script that uses some functions of the Octave Image package v.2.12 [63].

**Figure 2:**
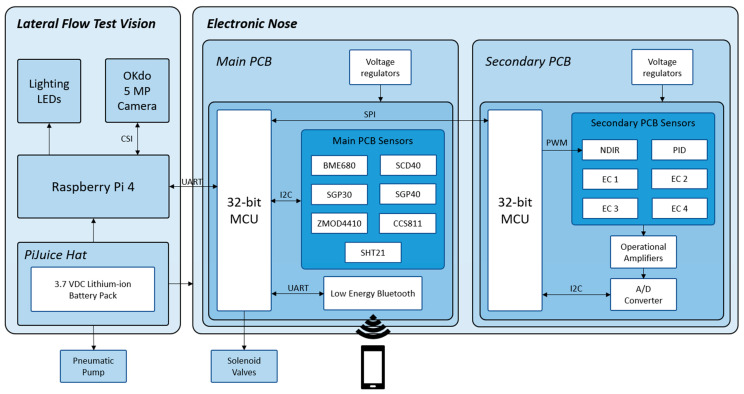
Block diagram of the TOMATO-NOSE.

The block diagram of this whole electronic nose plus Raspberry Pi and camera system is shown in Figure 2.

#### 2.3.4. Holder Cassette Design

A holder cassette was designed to hold the previously selected Botrytis cinerea lateral flow test because the manufacturer provides them without a holder. This holder was designed by Autodesk Inventor Professional 2023.3 software and printed by the Creality Ender3 V1 device, with a white 1.75 mm filament of 3D FDM PLA material (RS Amidata ref: 832-0223). The dimensions of the cassette are 22 × 88.9 × 7.1 mm (see Figure 1b). It is planned to save a QR code sticker down the test window to obtain information for the reader about the type of test, expiration time, and other data. Also, a triangle-shaped marking has been added to indicate the orientation in which the cassettes should be inserted into the chamber housing.

#### 2.3.5. Pneumatics Description

For the pneumatic part of the prototype, there are two S070C-RBG-32 solenoid valves from SMC Corp. (Tokyo, Japan), which have three ports and two positions in a 3/2 configuration. For the pneumatic pump, model 20020304 of the 2002 series from Gardner Denver Thomas GmbH (Fürstenfeldbruck, Germany) was chosen. It is a pneumatic diaphragm pump with a maximum flow rate of 380 mL/min that can be powered by 3.5 V_DC_ to 7 V_DC_.

The sensors are enclosed in a cell that is screwed to both PCBs and has inlet and outlet fittings to allow the flow of gases. The junction of the cell with the PCBs is made with gaskets to ensure the cell is watertight. It has been ensured that the number of vias is kept to a minimum in the area where the cell is placed, and they are also covered to prevent leaks.

**Figure 3:**
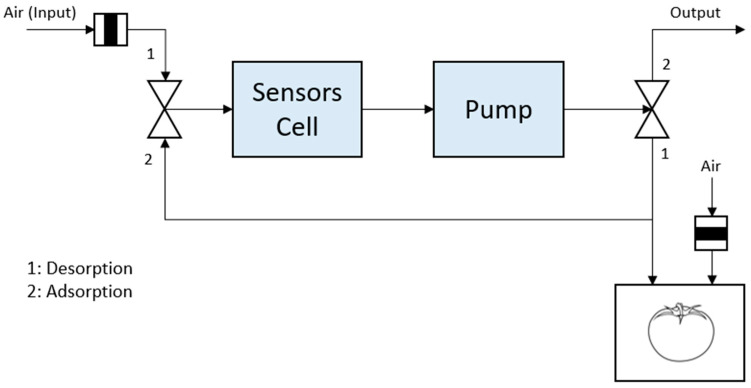
Pneumatic connections diagram of the TOMATO-NOSE.

The connection diagram of the pneumatic part is shown in Figure 3.

This diagram shows how the two solenoid valves are used to draw air in and out of the bag with a single pump. Both solenoid valves are synchronized and change state together. When the experiment is in the desorption phase, the clean air (or reference air) passes through an activated carbon filter and is introduced into the gas cell through the first solenoid valve. From there, it is sucked in by the pump and passes into the bag containing the tomato to fill it with fresh air. At the end of the desorption time, both solenoid valves switch simultaneously, and the gas now passes from the bag into the gas cell (it cannot return through the second solenoid valve). In the cell, it is sucked in again by the pump, and, this time, this air is expelled to the outside through the second solenoid valve. To prevent a vacuum from forming in the bag during this process, an external air inlet can be placed in the bag itself. In this case, it would be necessary to install another air filter to prevent unwanted odors from entering the bag.

#### 2.3.6. Prototype External Design

**Figure 4:**
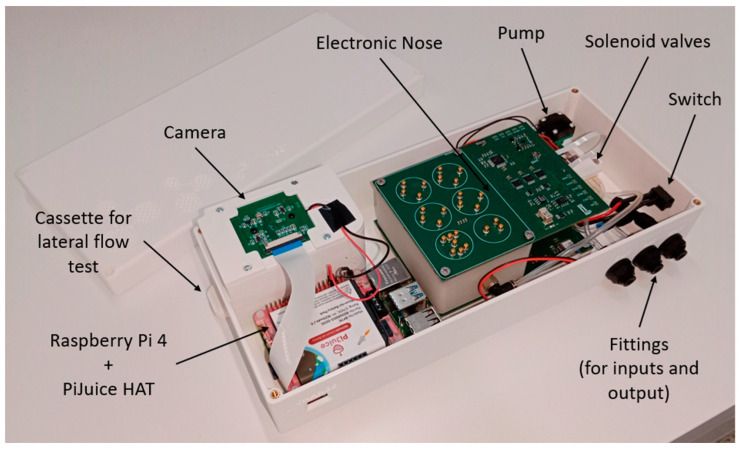
TOMATO-NOSE photo, with the arrangement of each of its parts.

This entire system is embedded in a 3D-printed housing measuring 26.65 × 12 × 5.9 cm, which has a small hole to access the micro-USB connector of the PiJuice HAT, some fittings (to connect the bag with the tomatoes, the reference air inlet, and the gas outlet), the hole through which the cassette is inserted into the chamber housing, and a switch to turn the device on and off. The layout inside this housing is shown in Figure 4.

#### 2.3.7. Image Processing

The image processing module splits into two blocks or parts: the location of the test window and the analysis of the test window content.

**Figure 5:**
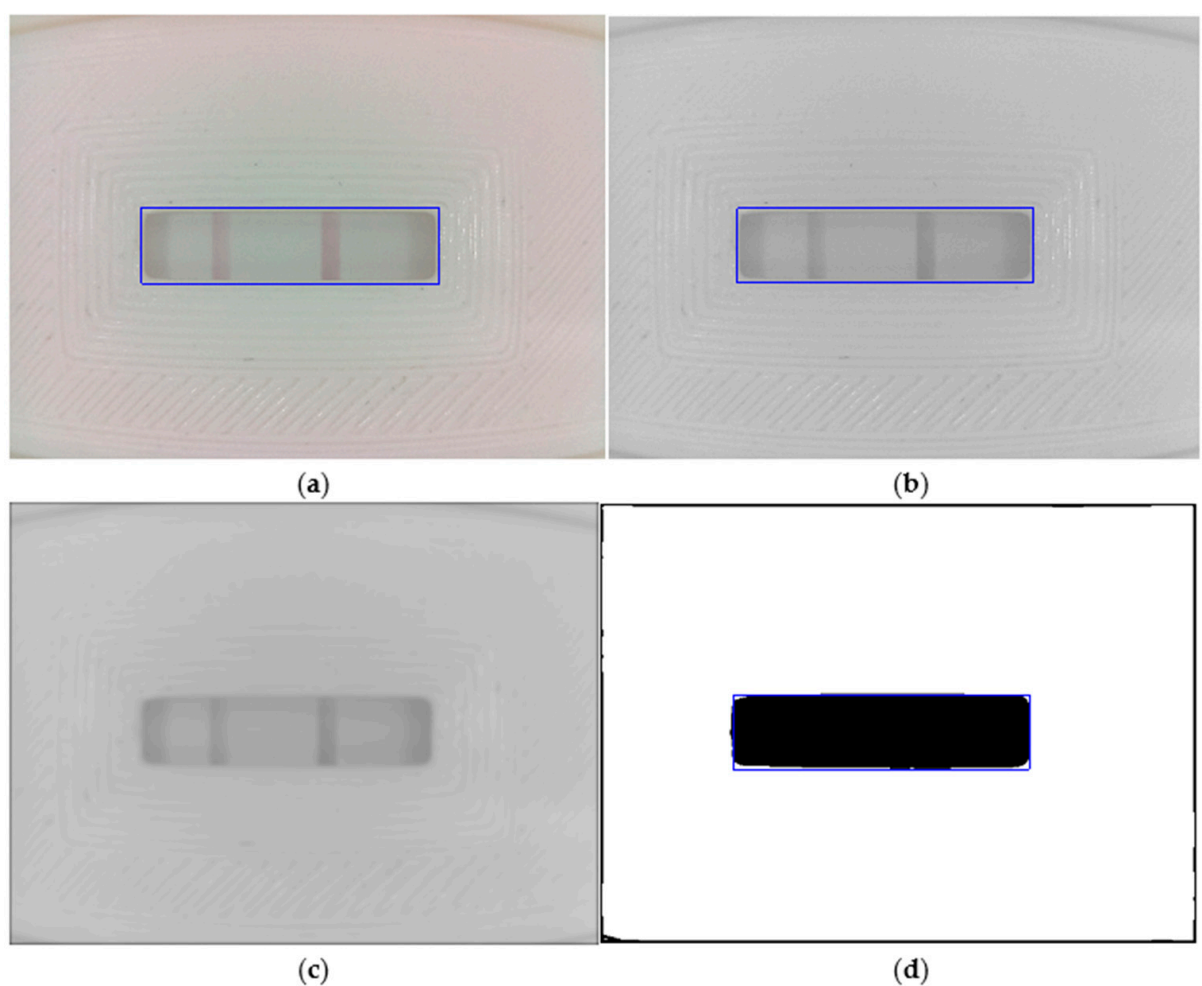
Image processing module: (**a**) 24-bit RGB input with test (left) and control (right) bars; (**b**) 8-bit grayscale version of (**a**); (**c**) Gaussian smoothing of (**b**) (sigma = 10); (**d**) Otsu binarization of (**c**).

In the first part, the acquired image is processed to segment the test window from the rest of the cassette. This allows detection to be less dependent on the relative position and alignment of the camera concerning the test device. It is achieved primarily by low-pass filtering and thresholding a grayscale (i.e., intensity) version of the input image (see Figure 5).

**Figure 6:**
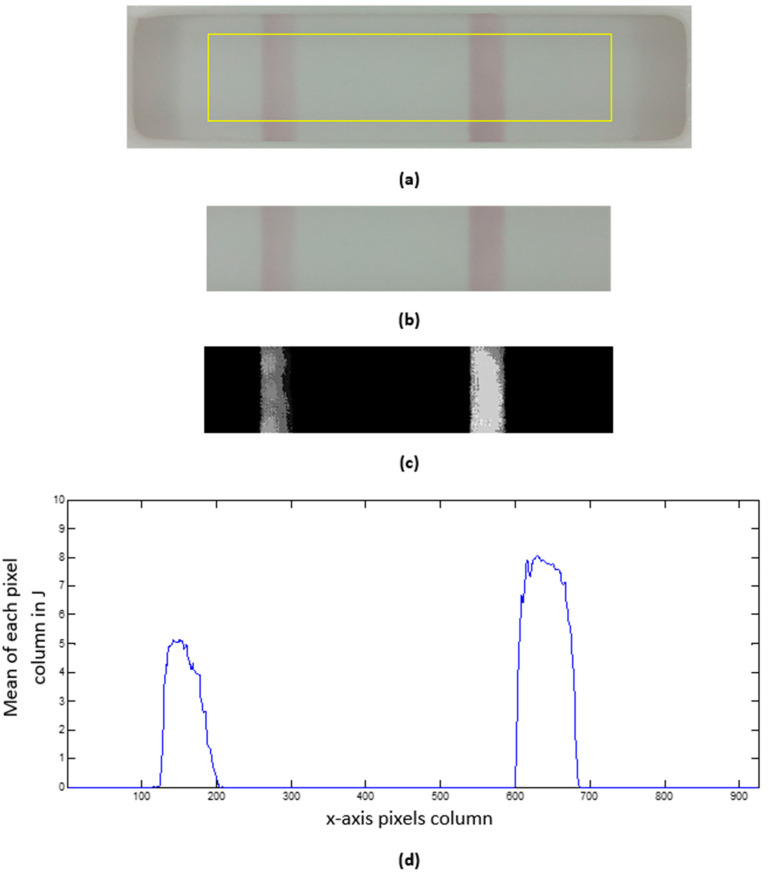
(**a**) Output of Part 1 (PNG, 1294 × 330 px), with test (left) and control (right) bars; (**b**) 5/7 crop; (**c**) background subtraction (contrast-stretched for viewing purposes); (**d**) mean profile.

**Figure 7:**
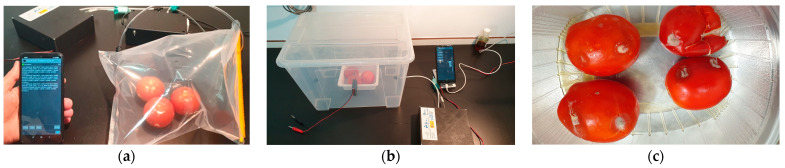
(**a**) Evaluation of the olfactory pattern of healthy tomatoes using an airtight bag, (**b**) evaluation of the olfactory pattern of non-healthy tomatoes in a rigid plastic container; (**c**) tomatoes infected with *B. cinerea*.

**Figure 8:**
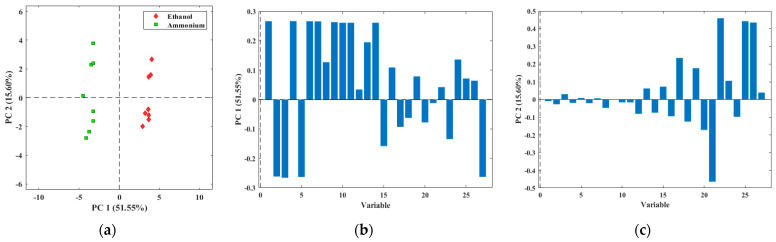
(**a**) PCA of the measurements of 5% ethanol and 5% ammonia solutions; (**b**) PCA loading plot for PC1; (**c**) PCA loading plot for PC2.

A more detailed description follows:

1. Color transformation. From 24-bit RGB (Figure 5a) to 8-bit grayscale (Figure 5b).
2. Gaussian smoothing. The image is low-pass-filtered to reduce bright and dark spots that appear on the textured surface of the test cassette (Figure 5c). We use a 2D Gaussian filter with sigma = 10.
3. Automatic image binarization via the Otsu thresholding algorithm (Figure 5d) [63].
4. Window location. As Figure 5d shows, the binarized image separates the test window area (in black) from the cassette. However, to safely locate the window sub-image, we first reconstruct the black border area, which is a product of the Gaussian filtering stage. Next, we compute the horizontal and vertical sum arrays from the reconstructed binary image. Finally, we select the window sub-image as those rows (columns) with horizontal (vertical) sum array values less than the mean value of that array. The resulting test window sub-image (1294 × 330 px) is shown in Figure 5a–d as a blue rectangle overlaid on each image.

The second part of the image processing module is devoted to interpreting the visual content of the test window as extracted from the camera input in Part 1. Our focus here is to obtain a more compact representation of the test window as follows (see Figure 6):

1. Cropping. The resulting image from Part 1 is a safely cropped sub-image where the test window is entirely preserved, but small portions of the cassette are regularly included at its border. While this sub-image may be useful for data logging, a further cropped image is more beneficial for data analysis when cropping is used to reject outliers. As Figure 6a shows, we discard the top and bottom fifths (1/5) of the image height and the left and right sevenths (1/7) of the image width to remove the cassette’s edge pixels and the lateral shadows it projects onto the test window. Thus, for the depicted example of size 1294 × 330 px, the centrally cropped sub-image (yellow rectangle in Figure 6a) has dimensions of 926 × 199 px.
2. Background subtraction. The selected chromatography lateral flow test technique allows two visible bars to appear in the previous test window cropped image (Figure 6b). These bars are called the control and test lines, and our properly white-balanced camera perceives them as slightly red pixels on a mostly gray background. If the control line is not visible, the test is null; otherwise, the test is positive only if the test line appears in the image. To further facilitate analysis, we transform the cropped RGB image into an 8-bit grayscale image (J) by subtracting the green (G) channel from the red (R) channel. Figure 6c shows a contrast-stretched version of J for the represented example, with discernible test (left) and control (right) bars, showcasing the benefits of the method.
3. Test profile. As a means of data reduction, we finally obtain a test profile array P that is computed as the mean of each pixel column in J (see Figure 6d). In this way, P is a more compact representation of the test image, allowing a straightforward analysis of the strength of the perceived test bars that can be characterized by, e.g., the local maximum of each peak in P.

#### 2.3.8. Communication Protocol

The TOMATO-NOSE is operated via a smartphone via Bluetooth. As indicated in Section 2.3.1, the main board consists of a Bluetooth module that communicates with the microcontroller via transparent UART. The microcontroller on this board acts as the main microcontroller, as it is the one that communicates with the secondary board, the Raspberry Pi 4, and the smartphone.

When the device is turned on, the microcontrollers on both boards (hereafter referred to as the main microcontroller for the main board and the secondary microcontroller for the secondary board) perform an initial configuration and then keep the sensors warm (to make good measurements). At the same time, the main microcontroller waits to receive a message from both Bluetooth and the Raspberry Pi 4, and the secondary microcontroller waits to receive communication from the main microcontroller. When the Raspberry Pi 4 is turned on, it automatically executes a Python code that configures the serial port and then waits for a message from the main microcontroller.

Communication between the smartphone and the main microcontroller is carried out via ASCII-based commands. These commands allow to start or stop sending data from the gas sensors, start the acquisition and processing of the lateral flow test image, and change some configuration parameters, among others.

To receive all the data on the smartphone, an application was developed for Android operating systems, which acts as a terminal and allows the data to be stored in a text file in a folder on the smartphone for subsequent processing on a computer. This application has an intuitive user interface that allows the use of the electronic nose without the need to know the commands.

### 2.4. Experimental Procedure

#### 2.4.1. Laboratory Test

To test the correct functioning of the olfactory part of the TOMATO-NOSE, a laboratory experiment was carried out with a 5% solution of ethanol in water and a 5% solution of ammonia in water. For each solution, 8 cycles of 1 min of reference clean air measurements (desorption) and 1 min of sample measurements (adsorption) were carried out, giving a total of 16 min of measurements. Since the device takes measurements every 2 s, a total of 480 measurements were taken for each solution.

#### 2.4.2. Tomato Analysis with the Electronic Nose

Tomato samples at stage 4 of maturity (pink color of the fruit between 30% and 60% of its surface) were selected for the analysis. The tomatoes were washed by immersion for 30 s in a 200 mg·L^−1^ sodium hypochlorite solution, dried, and refrigerated at 5 °C until analysis.

The color of the epidermis is a good indicator of the maturity stage of the tomato. The number of days required to reach maximum ripeness, when more than 90% of the surface is red, depends on the storage temperature and the degree of ripeness at the cutting time. The increase in temperature favors the rapid ripening of tomatoes with a shelf life of about three days at 25 °C [64].

The TOMATO-NOSE was switched on 15 min before analysis for sensor conditioning. The E-nose was set up with a sampling and cleaning time of 120 s each and cycled for 6–10 measurements per sample. Tests were carried out to study tomatoes’ olfactory pattern of maturity inside a bag and/or rigid plastic container. In addition, the olfactory pattern of tomatoes infected with *B. cinerea* was studied. For this, the tests carried out were the (I) evaluation of the olfactory pattern of healthy tomatoes in an airtight bag, (II) evaluation of the olfactory pattern of non-healthy tomatoes in a rigid plastic container, and (III) early detection of tomatoes infected with *B. cinerea*. Data obtained by TOMATO-NOSE were subjected to an exploratory unsupervised Principal Component Analysis (PCA).

(I) In the first test (Figure 7a), three healthy stage 4 tomatoes were placed in airtight bags for 20 min at 25 °C, sufficient time for the tomato VOCs to migrate into the headspace of the bag. Then, with the help of a pump, air was introduced through a valve placed in the bag so that no vacuum was generated when the air was sucked out of the bag for the E-nose measurements. At the end of data collection, the tomatoes were removed from the bag and left at about 25 °C to accelerate ripening. After three days, the same process was repeated with the tomatoes at stages 5–6.

(II) In the second test (Figure 7b), the three tomatoes from the first trial were incised to accelerate deterioration. They were placed in a rigid plastic container of about 15 L for three days. The container had an air inlet hole and another for sample collection. The two tomatoes were placed inside the container for three days and 10 measurements were taken per day with 1-hour intervals between each measurement.

(III) In the third test (Figure 7c), red tomatoes were taken from the refrigerator and groups of 4 tomatoes were kept in three plastic containers at 25 °C for six days. They were subjected to the following treatments: T0: healthy tomatoes without any infection; T1: tomatoes with an incision in the fruit with material previously sterilized to generate a wound in the fruit; T2: tomatoes with an incision in the fruit with material previously sterilized to create a wound in the fruit and inoculated with *B. cinerea*.

## 3. Results

### 3.1. Laboratory Test Results

A PCA has been performed with the measurements of 5% ethanol and 5% ammonia solutions explained in Section 2.3.1. As shown in Figure 8a, the E-nose can differentiate between the two solutions, with a variance of 52% for the first component (PC1) and 16% for the second component (PC2).

In addition, the loading plots of the principal components of the PCA have been obtained and are shown in Figure 8b for PC1 and Figure 8c for PC2. These plots represent each sensor signal’s load or influence on each principal component. The height of each bar represents how much it influences the principal component. That is, the higher they are, the more influence they have, and the lower they are (or close to 0), the less influence they have. When two or more signals have the same height, it means that both are redundant, so ideally, each should have different heights.

**Figure 9:**
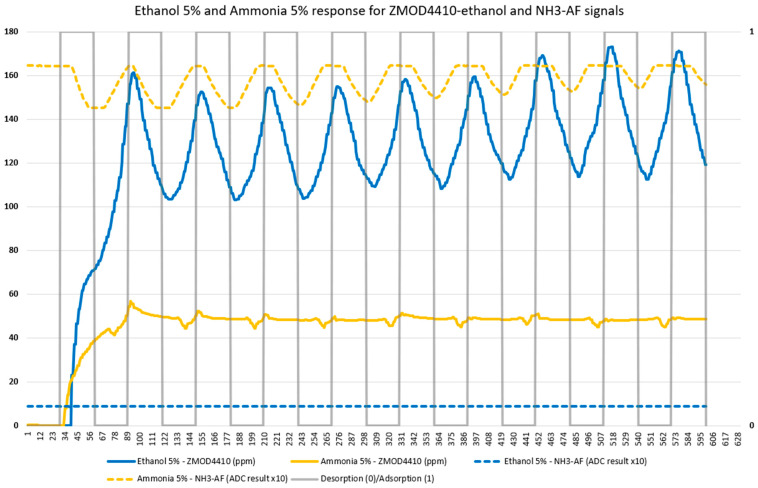
ZMOD4410-Ethanol signals and NH3-AF signals for ethanol 5% and ammonia 5%.

The difference observed in the results between the two compounds is because the sensors react differently to both compounds. In addition, there is a specific electrochemical sensor for ammonia (NH3-AF) that responds to this compound and does not react to others, such as ethanol. There are also MOS sensors that give ethanol signals (SGP30 and ZMOD4410) that are different for both compounds. Figure 9 shows the ethanol signal from the ZMOD4410 sensor and the NH3-AF sensor signal for the two compounds. The blue lines correspond to the 5% ethanol measurements and the yellow lines correspond to the 5% ammonia measurements. The continuous lines belong to the ZMOD4410 sensor and the dashed lines belong to the NH3-AF sensor. The gray line indicates 0 when the nose is in the desorption phase and 1 when it is in the adsorption phase (secondary axis on the right).

This graph shows how two sensors react differently to two different compounds.

Ethanol sensors based on MOS materials have been widely studied. The conductivity type of MOS is easily changed by the surrounding atmosphere (oxidizing gas or reducing gas). Therefore, MOS-based electrochemical sensors for ethanol have received much attention. MOS ethanol sensors are widely used to detect the presence of ethanol in food quality testing [65].

Electrochemical sensors, such as ammonia sensors, detect the presence of gas through a membrane containing electrodes separated by an electrolytic cap, which oxidizes in contact with the gas and emits an electrical signal due to the polarization of the electrodes. This type of sensor is used in the atmosphere with humidity changes [66].

### 3.2. Evaluation of Tomato Olfactory Pattern with TOMATO-NOSE

**Figure 10:**
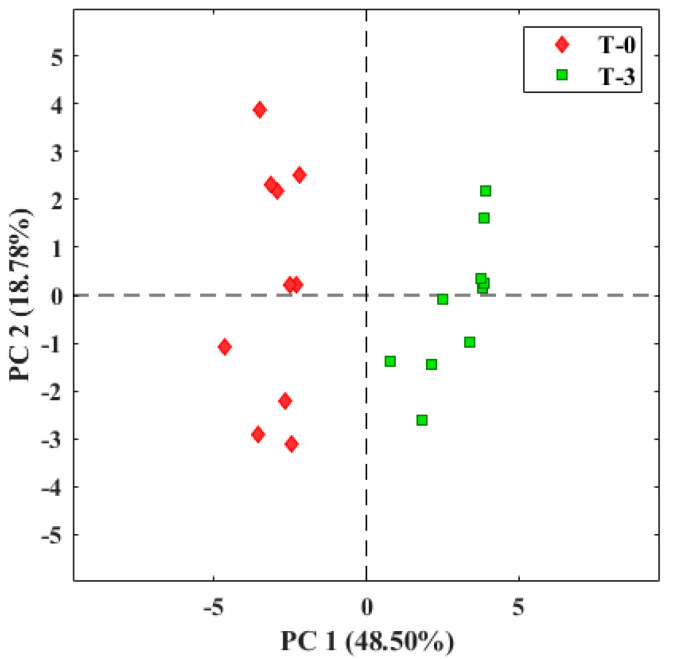
E-nose analysis of tomatoes placed in bags at 25 °C. T-0: tomato on day zero; T-3: tomato on day three.

The information obtained from the E-nose presents multiple variables, one for each sensor. To interpret them, it is necessary to reduce them as much as possible. For this, PCA was used [67,68,69]. In the first test analyzing tomatoes in airtight bags, the PCA based on the first two components was able to differentiate between the olfactory pattern of healthy tomatoes analyzed in bags at day zero (stage 4 of maturity) and after three days of ripening (stages 5–6 of maturity) (Figure 10). 

Between the two components, 67.2% of the total variance of the data was explained. The red dots represent the ten measurements made on the tomato at day zero, which are discriminated from the green dots that correspond to the ten measurements made on the tomato after three days of ripening at 25 °C. Two classification groups of samples are moved from left to right in the different quadrants (Figure 10). This classification indicates that the samples have different characteristic aromatic profiles that allow the sensors of the TOMATO–NOSE to react with the aromas of each sample. The result showed that the TOMATO-NOSE was able to differentiate different maturity stages of the tomato.

Although the use of the bags gave good results in the TOMATO E-NOSE-nose analysis, they did not have a fixed volume of air and it was necessary to control the volume of air introduced into the bag using a flow meter and a stopwatch. Uncertainty due to differences in the air volume in the bags could detract from the repeatability of the measurements due to the greater or lesser dilution effect of the tomato VOCs inside the bag. For this reason, it was decided to compare the data obtained with the bags using a rigid container with an ambient air inlet hole to avoid creating a vacuum where the same volume of air was always retained and using injured tomatoes.

**Figure 11:**
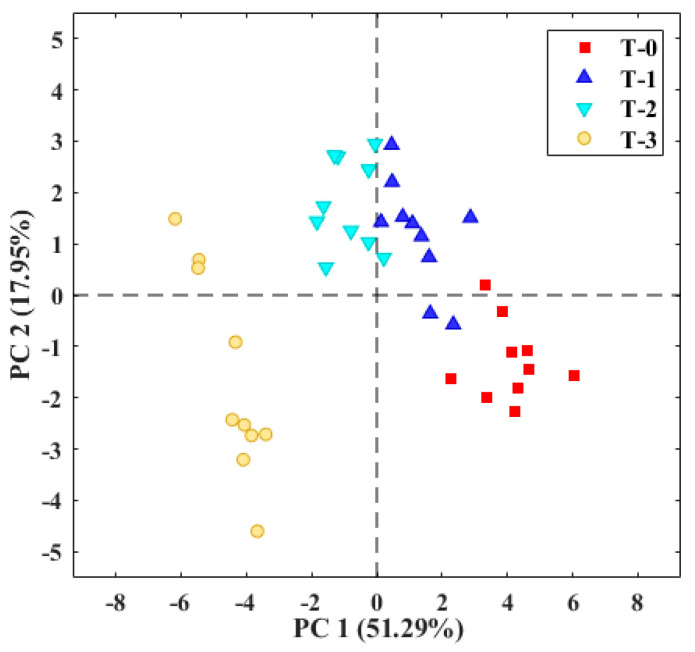
E-nose analysis of tomatoes placed for three days in a plastic container at 25 °C. T-0: tomato on day zero; T-1: tomato on day one; T-2: tomato on day two; T-3: tomato on day three.

The results of the second test, with the application of TOMATO-NOSE and tomatoes in a rigid container, are shown in Figure 11. 

The red dots from the first 10 h of measurements are at the bottom right, and as the tomato rot progresses, the groups of dots move further apart. During the post-harvest, the profile of volatile compounds of the tomato aroma varies daily with its maturation metabolism and storage conditions [4]. For this reason, this trend of distance between the groups of the different days is observed in the PCA graph. The use of the rigid plastic container reduced the uncertainty of the volume of the container. The variance explained by the first two principal components was 69.2%, slightly higher than the 67.2% obtained with the bags, and in both cases, sufficient to consider the results of the exploratory analysis adequate.

### 3.3. Botrytis Cinerea Infection

**Figure 12:**
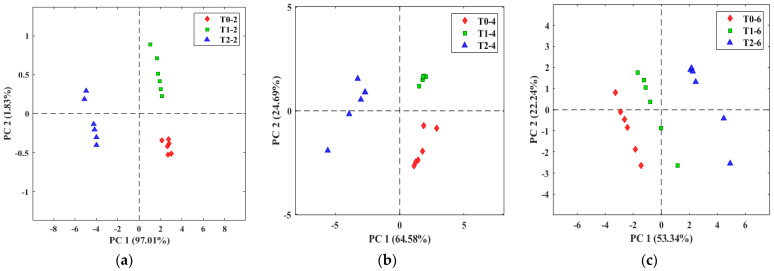
PCA of healthy tomatoes (T0) with rot (T1) and infected with *B. cinerea* (T2) on day 2 (**a**), day 4 (**b**), and day 6 (**c**) at 25 °C.

Tomatoes from stage 5 were infected with *B. cinerea* following Section 2.2 and take TOMATO-NOSE measurements from day 0 to 6 days. The infections were confirmed by microscopy. Figure 12 shows that the E-nose can detect differences between healthy, injured, and *B. cinerea*-infected tomatoes from two days after infection and before the fungus could be visible to the human eye. The PCA explained 98.8%, 89.3%, and 75.58% of the total data variance for days 2, 4, and 6, respectively. Therefore, in the three graphs, there is clear discrimination between the groups of measurements of the different treatments, perfectly identifying the group of values corresponding to the tomatoes infected with *B. cinerea* both in early (2 days) (non-visible fungus) and in advanced (6 days) infection (visible fungus). Therefore, these results could be used for the early detection of *Botrytis cinerea* in tomatoes.

### 3.4. Lateral Flow Test Device

#### 3.4.1. Commercial Lateral Flow Test Selection

Lateral flow devices (LFDs) have been used in other systems to detect and analyze fungal antigens, so it was thought that perhaps they could be used to detect levels of *Botrytis* antigens in finished wines, which could then be correlated with levels of off-flavors or desired flavors and aromas in the wine. In terms of application, LFDs are very easy to use and very fast, taking only 5–15 min and easily used by untrained workers in any workspace (no laboratory required). The formation of control and test lines shows positive results. Therefore, if a control line appears but not the second line (test line), it shows a negative result. The control line indicates whether the test has been performed correctly. Therefore, a null test could be obtained when the control line does not appear but the test line does.

Two commercial tests were tested for the selection of the most appropriate test for use in the TOMATO-NOSE: Botrytis Alert© (Mologic Ltd., Thurleigh, UK) and Loewe Fast Track© (LOEWE Biochemica GmbH., Sauerlach, Germany). Some of the characteristics that we value are high sensitivity (detection of low levels of *Botrytis* antigen in real samples to detect early infection) and specificity (only detection of *Botrytis* and no other fungus), intense test and control lines, and low detection time (5–10 min). Positive results are interpreted when, after contact with the sample diluted in the buffer, it reveals a second line or test line. Different test line intensities could be correlated with different B. cinerea concentrations. For this purpose, a battery of LFDs with positive and negative controls and real samples such as organic lemon and tomato infected with *Botrytis* (microscope analysis confirmed the presence of spores but not conidiophores (germination must be necessary)) were running. To avoid false results by saturation effects, serial dilutions (1/10, 1/100, 11,000) were performed and flowed in the strips. The formation of a control line will indicate that the test was run correctly. The assays with *Botrytis’s* control reagents and both LFDs showed correct results with positive and negative controls, but only Botrytis Alert’s LFD detected the *Botrytis* infection in vivo. For that reason, Botrytis Alert LFD was selected to develop the TOMATO-NOSE.

#### 3.4.2. Establishment of LFD Cut-Off Thresholds

The lateral flow cut-off is the limit of the amount of antigen (in this paper, *Botrytis cinerea* antigen) that will cause 50% of the test to be positive and 50% of the test to be negative. Therefore, for the LF reader, the cut-off threshold is the numerical pixel value of the test line where 50% of the tests are positive because a test line is visible and 50% of the tests are negative because no line is visible. Briefly, it is the limit amount to show negative or positive results. Above it, all tests must show positive results, and below it, negative results. We need to obtain the pixels that correlate with this cut-off to train and program the TOMATO-NOSE reader’s software v1.0. An LF test battery was run with positive, negative, and null controls at different concentrations to calculate it. This LF test battery was validated by human visual verification of the strips and using a smartphone web application provided by the manufacturer of these strips, checking that the result obtained by the device coincides with the two verification techniques. The manufacturer’s web application consists of taking a picture of the test with the smartphone camera and uploading it to the manufacturer’s server, where an algorithm determines the test result.

The selected test with a wide casuistry of test and control lines with different test and control line intensities shows the cut-off amount of positive control material. The null tests (no control line) were fixed with hot air and used to calculate peak values using the TOMATO-NOSE reader. These cut-off values establish positive and negative test limits. For that purpose, the TOMATO-NOSE camera captures an image of the LFD and proceeds, as in Section 2.3.7, to calculate the pixels. Table A1 shows the pixels of the control and test lines. The threshold values that have been established, according to the calibrations carried out with the photos taken, are 5% for the test line and 10% for the control line with respect to the maximum red color in each line.

#### 3.4.3. Vision Algorithm

Lateral flow test strips were used with tomatoes infected with Botrytis cinerea and the vision algorithm was tested with them. The result was satisfactory, with tests used on infected tomatoes being positive, tests used on non-infected tomatoes being negative, and tests not used being null. The validation of the vision algorithm is the same as for validating the results of the LF test battery used to establish the cut-off thresholds (Section 3.4.2).

**Figure 13:**
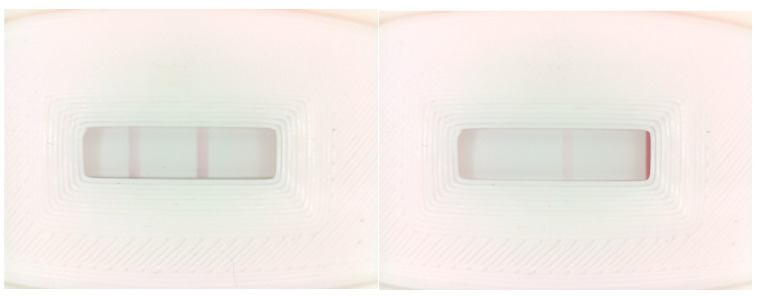
Photos taken by the device’s vision system.

The algorithm stores a history of all the photos taken, and some of these photos can be seen in Figure 13.

These results show that the vision part related to the lateral flow strips reinforces the result obtained by the olfactory part of the nose about a possible *Botrytis* infection in tomatoes.

## 4. Discussion

In this study, an E-nose, combined with a fast lateral flow test reader and a smartphone, was designed and trained to view results for the discrimination of tomato VOCS emitted at different maturity stages and putrefaction events. The device could be used by farmers in the crops and also by other stakeholders, such as post-harvest warehouse staff or store entrepreneurs. The E-nose device took only 15 min to capture and analyze the sample gases and several seconds to process the data. Although the E-nose technology is not generally capable of identifying specific compounds as a regular Mass Spectrometry (MS) system, it seems capable of differentiating mixtures of volatile compounds, thus allowing discriminations of VOC-releasing samples. The assays shown here demonstrate the effectiveness of the device for the detection of tomato maturity variables, one for each sensor, and to be able to interpret them, it is necessary to reduce them as much as possible. For that, we used PCA analysis. This type of analysis evaluates the ability of the sensor array of the E-nose to distinguish between different groups of gases and allow the graphical representation of the clustering of data with similar values. PCA has been previously used in E-nose applications for tomato quality assessment [67], tomato maturity assessment [68], early detection of microbial contamination in tomatoes [69], and fungal pests [70] with good discrimination results. However, it is necessary to identify the gases or VOCs by other techniques or, in this case, identify that the PCA differences detected by the E-nose correspond to *Botrytis cinerea* infection and not for other fungus. The TOMATO-NOSE is a useful tool for monitoring the health of the crop or storing tomatoes. Moreover, when the device detects the presence of *B. cinerea*, the lateral flow test could confirm by molecular antigens the nature of the fungus. As mentioned, the lateral flow reader is a convenient confirmatory and complementary tool to E-nose technology. However, further experimental trials with tomatoes should be conducted to optimize TOMATO-NOSE for in vivo use in crops and to identify the main VOCs detected during tomato lifespan and *B. cinerea* infection.

On the other hand, sample capture is one of the most critical points in making the device portable. For that, two different types of packaging were used to accumulate the VOCs of the tomatoes that were analyzed by the TOMATO-NOSE. Some authors used Teflon bags covering the tomato plant in their study of the development of a rapid E-nose system for early-stage diagnosis of tomato plants stressed by aphids in tomato analysis [55]. On the other hand, other authors used rigid containers in the use of E-noses for tomato quality assessment [67]. In both cases, as described in this work, the results were satisfactory. However, we prefer rigid containers to control the volume of air.

By deploying this new tool in crops and storehouses, farmers and markers will increase marketable yields, be able to monitor maturity status, reduce pesticide use and residue levels, and improve fruit shelf life.

Future assays with the TOMATO-NOSE and tomato plants in crops and greenhouses will be necessary to train the E-nose in the detection of specific VOCs of tomatoes and *B. cinerea* in real conditions. Complementary and parallel GC-MS assays on fruits are also recommended to identify specific VOCs during shelf-life and fungal infection.

## 5. Conclusions

This study successfully designed and tested a combined E-nose (with MOS and electrochemical sensors) and rapid test reader, named TOMATO-NOSE, for monitoring the volatile production of tomatoes during different ripeness stages and the early detection of *Botrytis cinerea* infection. The prototype demonstrated its effectiveness in discriminating the ripeness states of tomato fruit and detecting early pest agents in situ.

The E-nose component of TOMATO-NOSE demonstrated reliable performance in differentiating between 5% ethanol and 5% ammonia solutions, indicating its capability to discern different volatile compounds. The PCA analysis on tomato olfactory patterns further proved its ability to differentiate between the olfactory profiles of healthy tomatoes at different maturity stages. Furthermore, the TOMATO-NOSE effectively detected the presence of *Botrytis cinerea* infection in tomatoes. The E-nose measurements showed clear discrimination between healthy, injured, and *B. cinerea*-infected tomatoes, even before the fungus became visible to the human eye. The early detection of *B. cinerea* infection is crucial for preventing further spread and minimizing crop losses.

Overall, the TOMATO-NOSE prototype represents a powerful and farmer-friendly tool that could aid in the real-time monitoring of tomato ripeness stages and the early detection of pest agents. By providing accurate and timely information, this portable and low-cost device has the potential to enhance agricultural practices and optimize tomato production. Further research and validation in real field conditions are necessary before implementing the TOMATO-NOSE on a larger scale. Some shortcomings that need to be further investigated for improvement are the long data acquisition time, the large number of sensors, and the need to process the data on a computer, to give some examples. In this sense, improvements should be made to reduce the measurement acquisition time, eliminate sensors that have less influence on the results or are redundant, and have the device itself perform the data processing. Nonetheless, this study presents promising results and opens new avenues for the development of similar integrated sensing technologies for agricultural applications.

## Figures and Tables

**Table 1:** TOMATO-NOSE’s sensors.

Board	Sensor	Manufacturer	Type	Signals
Main Board	BME680	Bosch Sensortech GmbH, Germany	Metal Oxide (MOS)	Temperature, Relative humidity, Pressure, Resistance value
	SGP30	Sensirion AG, Switzerland	MOS	CO_2_, TVOCs ^1^, H_2_ (raw signal ^2^), Ethanol (raw signal)
	SGP40	Sensirion	MOS	Resistance value
	ZMOD4410	Renesas Electronic Corporation, Japan	MOS	Ethanol (raw signal), Resistance value, CO_2_, TVOCs, IAQ ^3^
	CCS811	ScioSense B.V., The Netherlands	MOS	CO_2_, TVOCs, Resistance value
	SHT21	Sensirion	MOS	Temperature, Relative humidity
	SCD40	Sensirion	Photoacoustic	CO_2_, Temperature, Resistance value
Secondary Board	IRM-AT	Alphasense Ltd., UK	Non-Dispersive Infra-Red (NDIR)	Reference electrode, Active electrode, thermistor output
	PID-AH2	Alphasense	Photo Ionization Detector (PID)	Raw output
	NH3-AF	Alphasense	Electrochemical (EC)	Work electrode, Active electrode
	ETO-A1	Alphasense	EC	Work electrode, Active electrode
	HCN-A1	Alphasense	EC	Work electrode, Active electrode
	SO2-AF	Alphasense	EC	Work electrode, Active electrode

^1^ Total volatile organic compounds. ^2^ Pre-processed signals from sensor resistance. ^3^ Air Quality Index.

## Data Availability

Data of sensors will be available upon request.

## Appendix A

**Table A1:** Test sample measurement and images using the image processing procedure of Section 2.

	J		P (Peak Values)		Ground Truth			Image
Picture	Min	Max	Test	Control	Test	Control	Class	T C
010730.PNG	0	10	5.1710	8.0450	Yes	Yes	Positive	
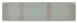
012623.PNG	0	14	0.3822	10.3418	No	Yes	Negative	
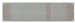
012735.PNG	0	15	3.9090	11.6835	Yes	Yes	Positive	
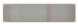
012842.PNG	0	14	0.2562	10.5070	No	Yes	Negative	
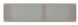
013013.PNG	0	10	4.8370	4.8320	Yes	Yes	Positive	
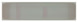
013126.PNG	0	3	0.0252	0.1860	No	No	Null	
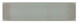
013225.PNG	0	14	8.5512	10.5056	Yes	Yes	Positive	
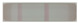
013409.PNG	0	12	8.7432	10.0104	Yes	Yes	Positive	
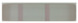
013457.PNG	0	8	4.0768	0.0456	Yes	No	Null	
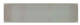
014230.PNG	0	14	8.9292	10.0310	Yes	Yes	Positive	
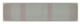
014415.PNG	0	16	12.3680	2.5504	Yes	Yes	Positive	
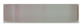
014923.PNG	0	9	5.0652	0.0000	Yes	No	Null	
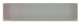
015023.PNG	0	14	0.6020	10.5000	No	Yes	Negative	
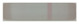
015144.PNG	0	8	0.0200	3.9904	No	Yes	Negative	
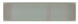
015300.PNG	0	12	9.3672	0.0612	Yes	No	Null	
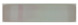
015437.PNG	0	8	4.8976	2.1120	Yes	Yes	Positive	
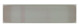
015537.PNG	0	5	0.0000	2.3170	No	Yes	Negative	
